# Identification of *SikCDPK* family genes to low-temperature by RNA-seq approaches and functional analysis of *SikCDPK1* in *Saussurea involucrata* (Kar. & Kir.)

**DOI:** 10.3389/fpls.2024.1436651

**Published:** 2024-09-30

**Authors:** Guangzhen Shi, Yuling Liu, Xiaohan Tian, Jiaxiu Guo, Xinxia Zhu

**Affiliations:** ^1^ Ministry of Education Key Laboratory of Xinjiang Phytomedicine Resource Utilization, Xinjiang Production and Construction Corps Key Laboratory of Oasis Town and Mountain-basin System Ecology, College of Life Sciences, Shihezi University, Shihezi, Xinjiang, China; ^2^ Xinjiang Second Medical College, Karamay, Xinjiang, China

**Keywords:** low temperature, *SikCDPK1*, expression analysis, functional exploration, calcium-dependent protein kinases (CDPKs)

## Abstract

Calcium-Dependent Protein Kinases (CDPKs) are a class of serine/threonine protein kinases encoded by several gene families that play key roles in biotic and abiotic stresses response and plant growth and development. However, snow lotus (*Saussurea involucrata* kar L.) CDPKs has rarely been reported. In this study, 20 *CDPK* genes in snow lotus were identified based on transcriptome data and classified into four groups (I-IV) based on their structural features and phylogenetic analyses. Among them, the transcript levels of *SikCDPK1* were significantly induced by low temperature and multiple hormone treatments, and *SikCDPK1* gene was found to have different expression in snow lotus seeds, leaves, stems and roots. The full-length promoter activity of *SikCDPK1* gene was higher than that of the 5’ end deletion fragment, and the promoter fragment containing the low temperature inducing element had increased activation after low temperature treatment. The promoter activity of *SikCDPK1* gene was mainly expressed in roots and rosette leaves. In addition, overexpressing plants of *SikCDPK1* were more tolerant compared to the wild type after being subjected to low temperature stress. Physiological analyses indicated that *SikCDPK1* improved plant tolerance to low temperature stress by maintaining cell membrane stability and reducing the accumulation of reactive oxygen species (ROS). These findings provided insights into *CDPK* gene families in snow lotus and broaden our understanding of the biological role of *SikCDPK1* and the mechanism of low temperature stress tolerance in snow lotus.

## 1 Introduction

In plants, low temperature is a common environmental stress that has important effects on plant growth and development. To adapt to the complex and changing external environment, plants have evolved a series of regulatory mechanisms, in which the kinase signaling pathway plays an important role (Zhang et al., 2022). In past studies, an important class of plant kinase family, the *CDPK* family, has been identified, and members of the *CDPK* family play an important role in the regulation of a variety of biological processes, including low temperature response, through Ca^2+^ signaling (Yip Delormel and Boudsocq, 2019).

Ca^2+^ plays an irreplaceable role as an important and ubiquitous second messenger in plant growth and development as well as in biotic and abiotic stress response (Aldon et al., 2018). Indeed, calcium is both an essential plant mineral element and a secondary signaling molecule. It can keep calcium concentrations in the cytoplasm in the hundreds of nanomolar levels and outside the cell membrane in the 1-10 millimolar range (Luan and Wang, 2021). This natural concentration gap puts it in a Ca^2+^ transient state that can be used as a signal to rapidly sense external stimuli, which is also known as Ca^2+^ signatures (Allan et al., 2022).

CDPK is a specific calcium sensor in plants and typically contains four distinct structural domains connecting a Ser/Thr protein kinase structural domain to an EF-hands motif containing a Ca^2+^ binding structural domain flanked by *N*-terminal and *C*-terminal variable regions (Klimecka and Muszyńska, 2007). The VNTD structural domain is located at the *N*-terminal end of the CDPK and has a significantly different amino acid sequence and length. The amino acid sequences and lengths vary significantly, and most CDPKs contain *N*-myristoylation sites and S-palmitoylation sites, which have been linked not only to the subcellular localization of CDPK proteins but also to the specificity of substrate recognition (Hrabak et al., 2003; Asano et al., 2005; Stael et al., 2012; Asai et al., 2013). Upon binding Ca^2+^, CDPKs are activated by autophosphorylation and phosphorylation, and the active kinases phosphorylate downstream targets such as ions channel, transcription factors, and metabolic enzymes (Wernimont et al., 2010; Liese and Romeis, 2013).

Numerous studies have shown that members of the *CDPK* gene family are extensively involved in growth and development, biotic and abiotic stress response, and hormone metabolism in plants. CDPKs have been implicated in mediating Ca^2+^-regulated pollen tube growth as an important node in Ca^2+^ signaling pathways (Estruch et al., 1994; Moutinho et al., 1998). *IbCDPK28* expression levels increased significantly in sweet potato tuberous roots during tuberization, and may play a functional role in regulating tuberous root growth (Li et al., 2022). Cold-induced calcium signal is perceived by *AtCPK28* which in turn phosphorylates its downstream target *NLP7* thereby enhancing cold tolerance in plants (Ding et al., 2022). *VpCDPK9* and *VpCDPK13* enhance powdery mildew resistance by regulating SA and ethylene signaling *in vivo* (Hu et al., 2021). *AtCPK4* and *AtCPK11* are two important positive regulators in the ABA signaling pathway (Zhu et al., 2007). Maize *ZmCDPK1* plays a negative role in cold stress signaling (Mittal et al., 2017). Whereas *ZmCDPK4* positively regulates ABA signaling and enhances drought stress tolerance (Jiang et al., 2013). CDPKs are also involved in the synthesis as well as signaling of ethylene, auxin, jasmonic acid, gibberellin and ABA hormones (Li et al., 2017). Together, these studies suggest that CDPK-mediated abiotic stress and hormone responses are complex and conserved in plants.

Given their importance, the *CDPK* gene family has been widely identified and characterized in many plant species. For example, 34, 31, 30, 28, 29, 31, and 29 CDPKs were found in *Arabidopsis thaliana*, *Oryza sativa* (rice), *Brachypodium distachyon*, *Hordeum vulgare* (barley), *Setaria italica* (Foxtail millet), *Capsicum annuum* (pepper) and *Solanum lycopersicum* (tomato) (Cheng et al., 2002; Ray et al., 2007; Wen et al., 2020; Yang et al., 2017; Yu et al., 2018; Cai et al., 2015; Hu et al., 2016). Although a number of *CDPK* family genes involved in low temperature response have been identified, the understanding of the specific functions of these genes in low temperature adversity is still limited. Snow lotus is an excellent plant that tolerates extreme climates, and can grow in rock crevice in mountains and cliffs with perennial snow, rarefied air and strong ultraviolet radiation. The study of its CDPKs gene is undoubtedly of great significance for elucidating the low temperature tolerance mechanism of snow lotus.

However, up to now, there have been no reports of snow lotus CDPKs, and the functional studies of snow lotus *CDPK* genes are also rarely reported. In this study, we first identified 20 *SikCDPK* genes from the low temperature transcriptome database of snow lotus. The sequence, structure and evolution of SikCDPKs were analyzed by bioinformatics methods. The representative gene *SikCDPK1* was screened for cloning and expression analysis. The mechanism of *SikCDPK1* in low temperature response was also explored, which provided new clues to further reveal the mechanism of low temperature tolerance in snow lotus adversity.

## 2 Materials and methods

### 2.1 Screening and identification of *SikCDPK* gene family members involved in low temperature response

The snow lotus low temperature transcriptome database was obtained from (http://www.shengtingbiology.com/SaussureaKBase/index.jsp), meanwhile, the *Arabidopsis* CDPK family member protein sequences from the *Arabidopsis* Information Resource (TAIR, https://www.arabidopsis.org/) database. The seed files (PF00036 and PF07714) of the *CDPK* gene family were downloaded by the Pfam online database (http://pfam.xfam.org/), and the protein sequences containing the conserved domains of CDPK were analyzed by hmmsearch. All identified CDPK proteins of snow lotus were submitted to Pfam and NCBI website’s (Conserved Domain Database, CDD) for conserved structural domain validation to confirm the snow lotus *CDPK* gene family members.

### 2.2 Sequence analysis and subcellular localization of *SikCDPK* gene family members involved in low temperature response

The molecular weight, isoelectric point (pI), hydrophilicity index and other physicochemical properties of the snow lotus *CDPK* gene family were queried by the ProtParam tool (http://web.ExPasy.orP/ProtParam/) of the ExPASy online software; the online tool WoLF PSORT (https://wolfpsort.hgc.jp/) was used to analyze the sequence and subcellular localization of the *SikCDPK* gene family members. The online tool Myristoylaton (https://web.expasy.org/myristoylator/) was used to predict *N*-myristoylation, and CSS-Plam was used to predict palmitoylation. TMHMMServerv2.0 was used to predict the transmembrane structural domains, and Wolf POSRT tool (https://www.genscript.com/psort.html) was used for subcellular localization.

### 2.3 Phylogenetic tree construction of *SikCDPK* gene family involved in low temperature response

The protein sequences of snow lotus CDPK and *Arabidopsis* CDPK were compared and analyzed using MEGA 7.0 software and ClustalX, and the phylogenetic tree was constructed using the Neighbor-Joining algorithm with the Bootstrap value set to 1000.

### 2.4 Conserved motifs of the *SikCDPK* gene family involved in low temperature response

The conserved motifs of the CDPK family of snow lotus were analyzed using Multiple Em for Motif Elicitation (MEME, https://meme-suite.org/meme/tools/meme) online software, with the parameters set to a maximum discovery number of motifs of 10 and a maximum length of motifs of 100 nt (Nucleotide).

### 2.5 Cloning and bioinformatics analysis of *SikCDPK1* gene

According to the low temperature transcriptome sequencing data of snow lotus, the *GhCDPK1* gene sequence was used as a probe search, and after removing the duplicate sequences by multiple sequence comparison, the complete open reading frames (ORFs) of the sequences were analyzed one by one by using the ORF Finder tool (https://www.ncbi.nlm.nih.gov/orffinder) in NCBI. The ORF primers were designed in **Table 1**. The total RNA was extracted and the first strand of cDNA was synthesized according to the TaKaRa kit. The primers for the target genes, *SikCDPK1*-F and *SikCDPK1*-R, were used in **Table 1** for RT-PCR amplification, and the PCR program was as follows: pre-denaturation at 94°C for 5 min; denaturation at 94°C for 30 s; replication at 63°C for 40 s; extension at 72°C for 1 min and 30 s; 30 cycles. The positive product was named as *SikCDPK1*, sequenced by Beijing Huada Genetics, and the conserved domains of *SikCDPK1* gene were analyzed by SMART online analysis software (http://smart.embl-heidelberg.de/). The phylogenetic tree was constructed using ClustalX and MEGA7.0 software.

**Table 1:** The primers used in this study.

Primer name	Primer sequence (5’→3’)	Purpose
*SikCDPK1*-F	GGATCCATGGGGAATACTTGTGTTGGAC	Gene clone
*SikCDPK1*-R	GTCGACCCGTCGATACCGGAAAAAAC	Gene clone
*GAPDH*-F * GAPDH*-R * SikCDPK1*-qF * SikCDPK1*-qR	TAGCAAGGATGCTCCCATGTTCGT AAAGGAGCAAGGCAGTTGGTTGTC ATCCCAAACTGCCCTTGTCCTA GAAGATACCCCACCTACCCCTAAC	Internal genes primers Internal genes primers Gene expression analysis Gene expression analysis

### 2.6 Analysis of spatio-temporal expression pattern of *SikCDPK1* gene

The total RNA of snow lotus was extracted from the young stems and leaves of snow lotus grown for 45 days. Three biological repeats in each sample were performed. The first strand of cDNA was synthesized in accordance with the steps in the TaKaRa kit, and assayed by qRT-PCR. Amplification by qRT-PCR was performed using TB Green® Premix Ex Taq™ II (Takara). The qRT-PCR reaction was carried out with the primers in **Table 1** for the endogenous genes of GAPDH-F and GAPDH-R, as well as the primer for the target gene, *SikCDPK1*. Reaction system: cDNA (1 µL), *GAPDH*-F/*SikCDPK1*-qF (0.25 µL), *GAPDH*-R/*SikCDPK1*-qR (0.25 µL), SYBR Green PCR Master Mix (5 µL), ddH_2_O (3.5 µL), and the reaction program: 95°C pre-denaturation for 5 min; 95°C 10 s, 62°C 30 s, 72°C 30 s 40 cycles. Three replicates were set up for each sample, and the average value was taken. The expression of snow lotus *SikCDPK1* gene in different tissues and organs was calculated using the 2^-ΔΔCt^ method (Livak and Schmittgen, 2001), and the experimental data were subjected to LSD (least significance difference) multiple ANOVA using the SPSS 18.0 software, with the test of significance set at *P*<0.05 (significant level) and *P*<0.01 (highly significant level), and graphs were made in using Excel software.

### 2.7 Analysis of *SikCDPK1* gene involved in cold and hormone

The aseptic seedlings of snow lotus were placed in -4°C light incubator for cold stress treatment, and the leaf samples were taken from treatment 0, 1, 3, 6, 12, 24 h. Three replicates were set up for each sample, and qRT-PCR reaction was carried out in accordance with the above method, and the expression of *SikCDPK1* gene was calculated at different times of the low temperature treatment.

Snow lotus seedlings grown for 3 months with stable and healthy growth were selected, rinsed and placed in MS liquid medium separately. After 48 hours of recovery, they were placed in MS medium supplemented with 100 µM ABA, 100 µM GA_3_, 1 mM SA and treated for 0, 1, 3, 6, 12 and 24 hours, respectively. Samples were collected, and three replicates were set up for each sample. qRT-PCR reactions were performed according to the above methods. The expression of *SikCDPK1* gene after induction by low temperature, ABA, GA_3_, and SA was calculated by the 2^-ΔΔCt^ method (Livak and Schmittgen, 2001), and the experimental data were subjected to LSD using SPSS 18.0 software. Multiple ANOVA analysis was performed, and GraphPad Prism 9.1 was used for graphing.

### 2.8 Cloning and activity analysis of *SikCDPK1* gene promoter

**Figure 1:**
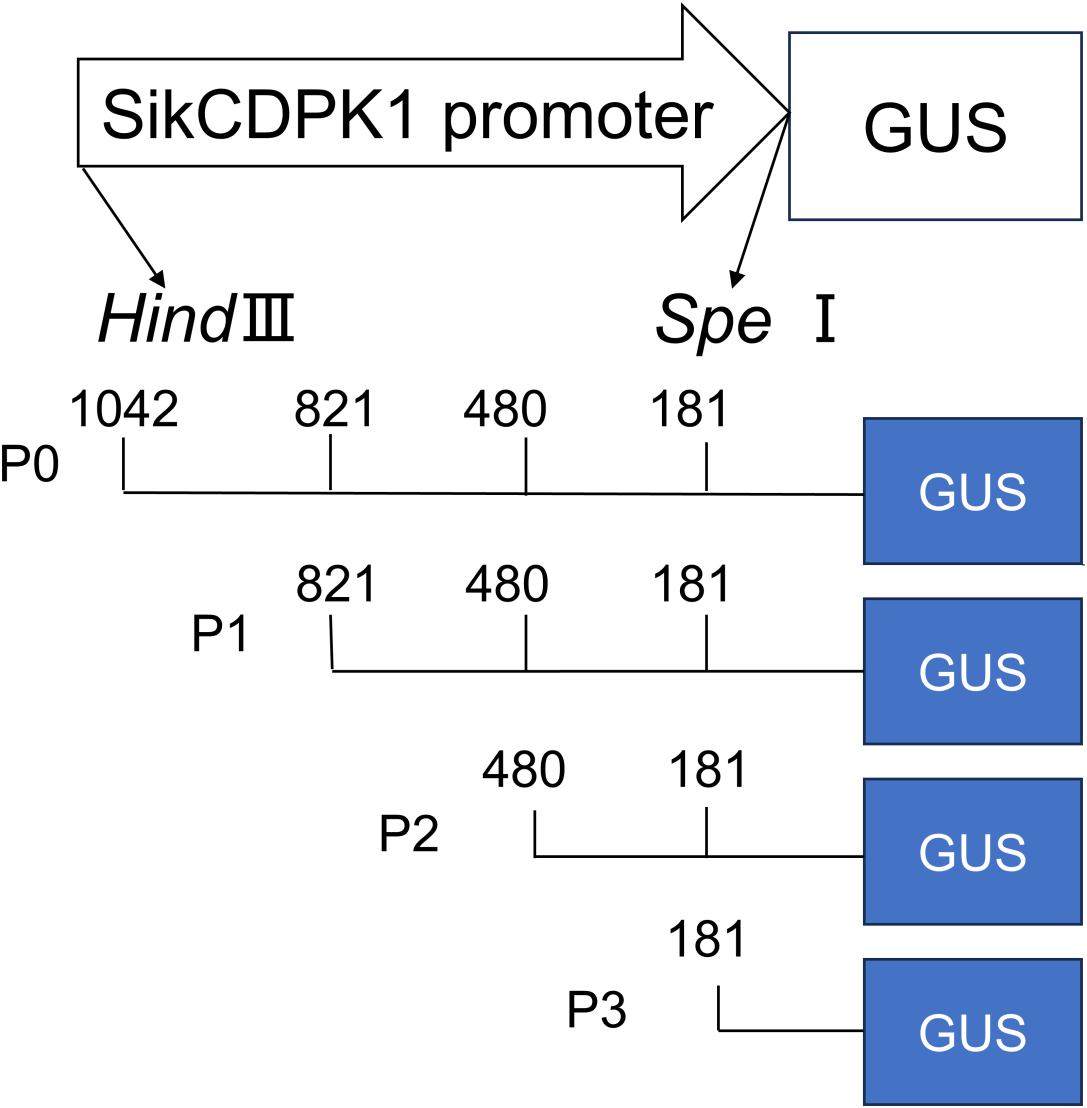
GUS expression constructs driven by 5′-deleted *SikCDPK1* promoters.

The *SikCDPK1* promoter was cloned from snow lotus by TAIL-PCR, and the cis-acting elements of the promoter region were analyzed using the promoter online analysis software PlantCare (http://bioinformatics.psb.ugent.be/webtools/plantcare/html/). The full-length promoter or 5’-end deletion promoter-driven GUS recombinant expression vector pCAMBIA1304-*SikCDPK1*-P0-GUS, P1-GUS, P2-GUS, and P3-GUS was constructed (**Figure 1**). *Arabidopsis thaliana* Col- 0 seeds were sterilized with 70% alcohol for 2 min and washed twice with sterile water; then sterilized with 2% NaClO for 7 min and washed with sterile water. The seeds were sterilized by 70% alcohol for 2 min and washed with sterile water for 2 times, then sterilized by adding 2% NaClO for 7 min and washed with sterile water for 5 times, and then spring-cleaned at 4°C for 3-4 d. The seeds were evenly sown on 1/2 of MS medium. They were incubated in a light incubator at 25°C for 16 h of light and 8 h of darkness, and then transplanted into pots containing vermiculite and nutrient soil (1:1 by volume) at the stage of 2-3 true leaves, and then cultured in an artificial climatic chamber under the same environmental conditions. Arabidopsis thaliana was genetically transformed by the flower drop method (Clough and Bent, 1998). After the transgenic *Arabidopsis thaliana* was screened to the purity, putting it in the low temperature (4°C). The P0-GUS pure transgenic *Arabidopsis thaliana* was used as a positive control and wild-type *Arabidopsis thaliana* (Col- 0) as a negative control, respectively. Mature leaves on the day of flowering were taken and immersed in 0.5 mg/L X- Gluc (Sigma) solution, and then decolorized with anhydrous ethanol to remove chlorophyll after being sheltered from light at 37°C overnight to observe whether blue color appeared or not, and photographed with a dissecting microscope (Vitha et al., 1995). The experiment was repeated three times independently. The promoter activity was analyzed in different tissues of *Arabidopsis thaliana* by GUS histochemical staining and low temperature treatment was analyzed.

### 2.9 Response of overexpressed *SikCDPK1* gene to cold stress

Using *Agrobacterium*-mediated method, pCAMBIA2300-GV3101-*SikCDPK1* was used to infest transformed tobacco leaf discs, and the screened positive trans-*SikCDPK1* gene tobacco was put into the artificial climate chamber at -4°C, and subjected to cold stress treatment for 0, 3, 6, 9 and 12h respectively, and then the plants were put into the incubator at 25°C to recover for 24h, and the morphological changes of the tobacco in the various treatments were observed. The plants were then put into the incubator at 25°C for 24h to observe the morphological changes of tobacco in different treatments, and the samples were taken at different treatment stages and stored in the freezer at -80°C. Three biological repeats in each treatment were performed.

### 2.10 Physiological indices measurements

To check the physiological indices changes, the leaves were used to identify relative electricity conductivity (REC), contents of MDA, after low temperatures treatments, whereas some plants were kept untreated as controls. Half a gram of fresh leaf sample (chopped into small pieces) was dipped in distilled water for half an hour, and REC1 was measured by using an REC meter. REC2 was recorded by heating the samples in a water bath at 90°C for 50 min. The final value of relative electricity conductivity (REC; %) was determined with this formula: REC%=(REC1÷REC2) ×100 (Chattha et al., 2022). Malondialdehyde (MDA) content was measured according to (Schmedes and Hølmer, 1989) with slight modifications. Powdered samples (0.2 g) were homogenized in 10 mL of 10% (w/v) trichloroacetic acid (TCA). Homogenate was centrifuged at 9660× g for 10 min. Then, 2 mL of the supernatant was mixed with 2 mL of 10% (w/v) TCA containing 0.6% (w/v) of thiobarbituric acid (TBA) and incubated at 100°C for 20 min, and then quickly cooled on ice followed by centrifugation at 9660× g for 10 min. Absorbance at 532, 600, and 450 nm was measured using Jenway 7305 UV/Visible Spectrometer (Jenway, London, UK). The MDA content was calculated according to the formula MDA content (µM/g) = 6.45 (OD _532_ − OD _600_) − 0.56 OD _450_. For the O^2-^ detection, detached leaves were immersed in 100 mL staining solution containing 0.1% (w/v) nitroblue tetrazolium (NBT), 10 mM sodium azide, 50 mM potassium phosphate, pH 6.4 for 15 min. After stopping the reaction with 95% ethanol, the samples were decolorized in 96% ethanol under heating at 40°C (Jambunathan, 2010). Superoxide ions react with NBT and appear as blue. These stained leaves can be photographed by light microscope.

## 3 Results

### 3.1 Identification and analysis of *SikCDPK* gene family involved in low temperature response

Using the snow lotus transcriptome as the reference sequence and HMMER 3.0 software to search for protein sequences containing the PF07714 model, and further analyzing the conserved structural domains by CDD and SMART software, a total of 20 SikCDPK family members were identified in the snow lotus transcriptome database (**Table 2**).

**Table 2:** Information of *CDPK* family in *Saussurea involucrata*.

ID	CDS(bp)	No. of aa	pI	Molecular weight(D)	GRAVY	Membrane	EFh	*N*-myristoylation	palm	Subcellular location
c10050_g1_i1	1545	514	5.41	57523.81	-0.354	no	4	no	yes	chlo
c72520_g1_i1	1605	534	6.03	59860.25	-0.487	no	4	no	yes	E.R
c72680_g1_i1	1563	520	7.22	58443.77	-0.476	no	4	no	yes	chlo
c72680_g1_i2	1512	503	6.31	56458.72	-0.429	no	4	yes	yes	chlo
c74106_g1_i1	1716	571	5.43	63495.27	-0.403	no	4	no	yes	cyto
c75351_g1_i1	1638	545	8.49	61420.79	-0.573	no	4	yes	yes	cyto
c75948_g1_i1	1353	450	7.13	51244.99	-0.368	no	1	yes	yes	cyto
c75948_g1_i2	1608	535	5.88	61546.42	-0.466	no	4	yes	yes	chlo
c76112_g1_i1	1638	545	6	61735.81	-0.403	no	4	yes	yes	cyto
c79943_g8_i1	1698	565	5.35	63174.64	-0.361	no	4	no	yes	chlo
c85443_g1_i1	1791	596	6.9	67115.63	-0.42	no	4	no	yes	nucl
c86489_g1_i1	1605	534	5.84	60157.54	-0.412	no	4	no	yes	cyto
c86489_g1_i2	927	308	5.07	34969.74	-0.487	no	4	no	yes	cyto
c86489_g1_i3	1602	533	5.83	60066.38	-0.459	no	4	no	yes	cyto
c86622_g2_i1	918	305	4.85	34471.36	-0.293	no	4	no	yes	chlo
c86622_g2_i2	1758	585	5.7	65052.96	-0.471	no	4	no	yes	cyto
c86680_g3_i3	1206	401	5.34	45628.68	-0.269	no	4	no	yes	cyto
c88390_g1_i1	1239	412	5.13	46752.45	-0.412	yes	4	no	yes	cyto
c118412_g1_i1	996	331	5.01	37376.69	-0.353	no	4	no	yes	cyto
c51437_g1_i1	1716	571	5.44	63524.35	-0.406	no	4	no	yes	cyto

The sequence lengths of the snow lotus CDPK family members ranged from 918 bp (c86622_g2_i1) to 1791 bp (c85443_g1_i1), encoding 305 ~ 596 amino acids, respectively, and the relative molecular weights of the proteins ranged from 34,471.36 D to 67,115.63 D. PI analyses revealed that the theoretical isoelectric point of 20 SikCDPK proteins were between 4.85 (c86622_g2_i1) to 8.49 (c75351_g1_i1), of which 17 SikCDPK proteins with pI < 7.0 (mean 5.62) were acidic proteins, and 3 SikCDPK proteins with pI > 7.0 (mean 7.61) were basic proteins. Analysis of GRAVY revealed that the average coefficient of hydrophilicity of all 20 SikCDPK proteins was less than 0, indicating that all snow lotus CDPK proteins are hydrophilic proteins. Analysis with TMHMM Server software revealed that only one SikCDPK protein (c88390_g1_i1) had a transmembrane structure, while the others did not have a transmembrane structural domain. EFh analysis of the calcium-binding region revealed that 20 SikCDPK family members have CDPK-typical, Ca^2+^-binding EF-hands structural domains, of which 19 family members contain four EF-hands structures like most CDPKs in other species, whereas one family member protein (c75948_g1_i1) has only one EF-hands structure. Analysis of *N*-myristoylation and palmitoylation revealed that 5 genes in the SikCDPKs family have *N*-myristoylation sites and all 20 have palmitoylation sites. Analysis of prediction subcellular localization revealed that 12 SikCDPK proteins were located in the cytoplasm, 6 SikCDPK proteins were localized on chloroplasts, 1 SikCDPK protein was localized in the endoplasmic reticulum, and 1 SikCDPK protein was localized in the nucleus (**Table 2**).

### 3.2 Phylogenetic analysis of snow lotus *SikCDPK* families involved in low temperature response

**Figure 2:**
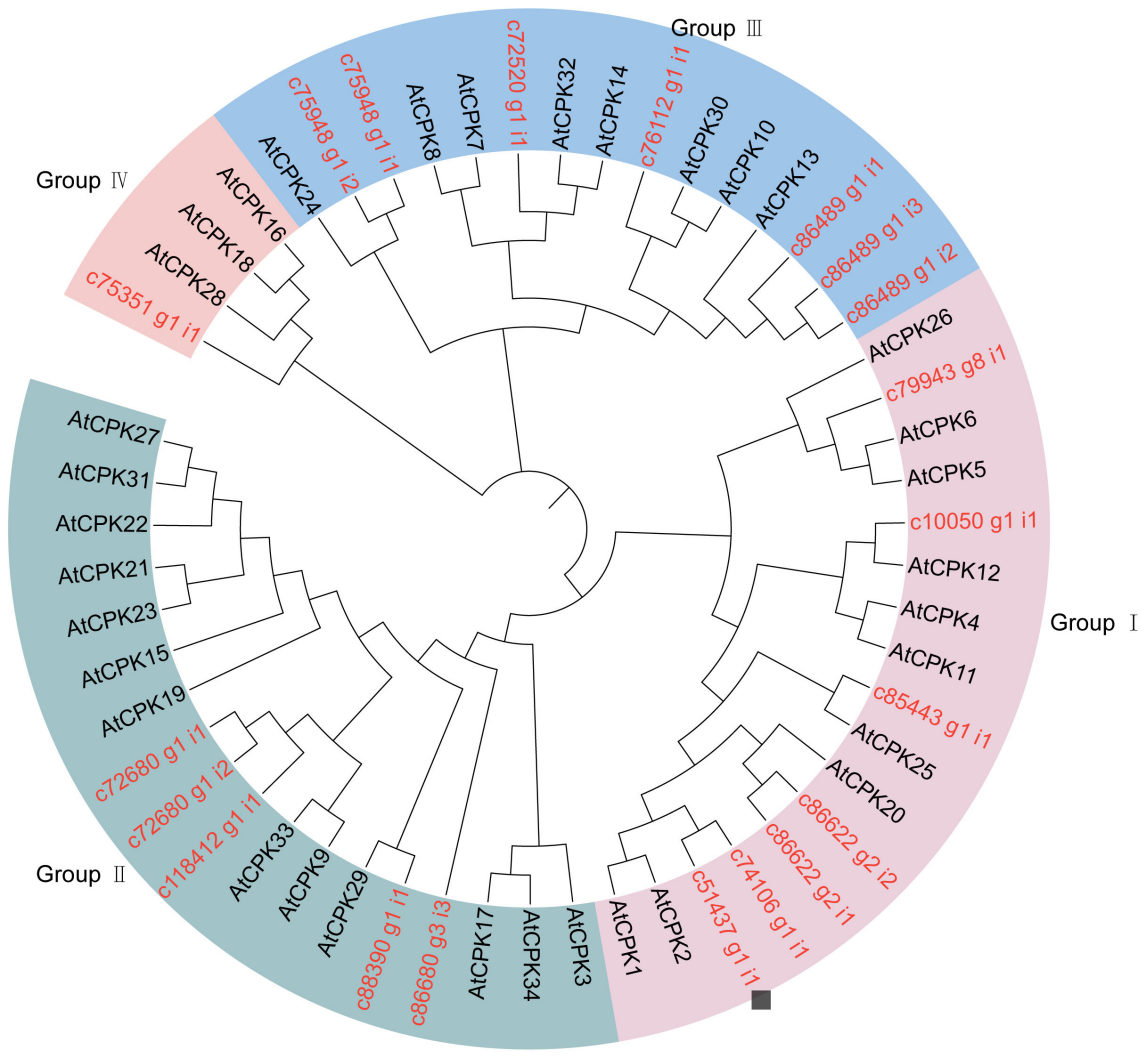
The evolutionary tree is composed of CDPKs from *Arabidopsis* and *Saussurea involucrate* The tree was generated using Mega 7.0 software by the Neighbor-joining method and bootstrap analysis (1,000 replicates) expressed in percentages. Members in the same group are clustered under the same color with a chromosomal location number ranging from; 20 SikCDPKs in snow lotus, and 34 AtCPKs in *Arabidopsis*.

In order to analyze the phylogenetic relationship of the *SikCDPK* gene family of snow lotus, in this study, the full-length protein sequences of 20 SikCDPKs and 34 AtCDPKs of *Arabidopsis thaliana* were imported into the MEGA 7.0 software, and the neighbor-joining method was used to construct a phylogenetic tree (**Figure 2**). With reference to the classification of *Arabidopsis thaliana*, the 20 SikCDPK proteins were classified into four subgroups, which were called subgroups of Group I, Group ll, Group III, and Group IV. Group I and Group III had the most members with 7 SikCDPK proteins, while Group IV subgroup had the least members with only 1 SikCDPK protein. An in-depth study of the clustering results of CDPK members revealed the presence of genes with similar evolutionary relationships in the same group, suggesting their homology.

### 3.3 Conserved motif analysis of snow lotus SikCDPK family involved in low temperature response

**Figure 3:**
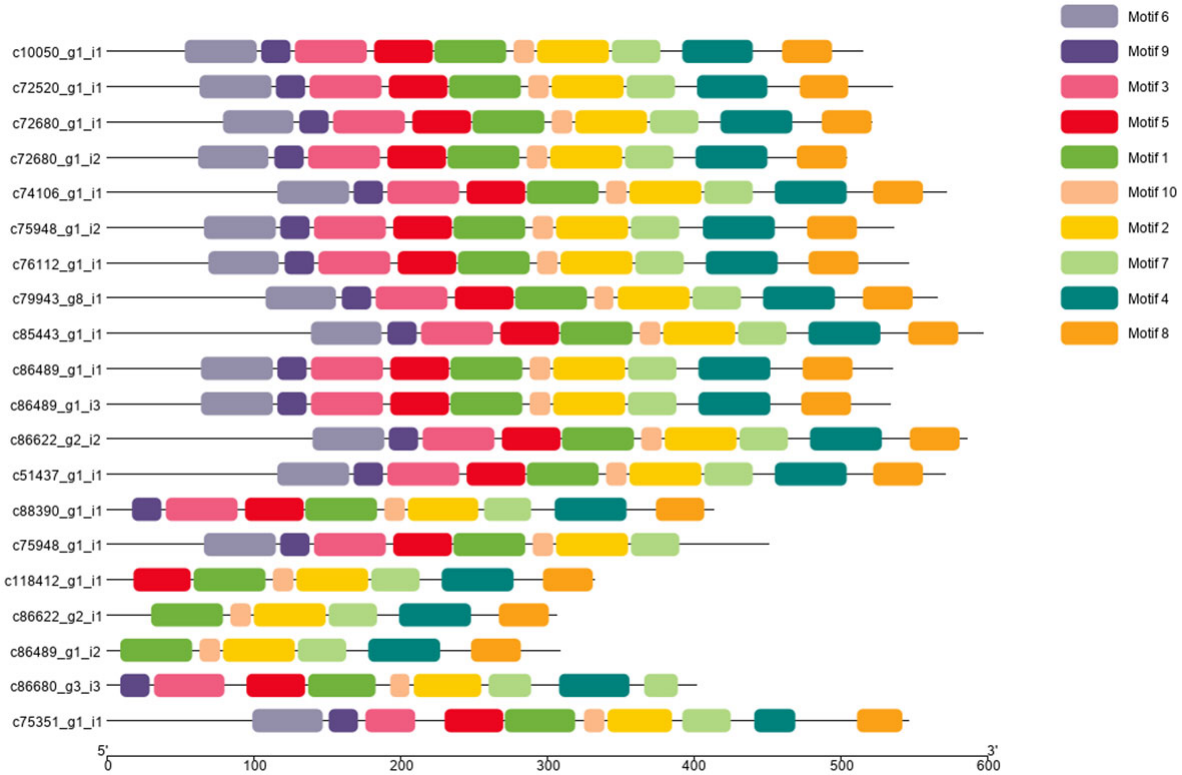
Conserved motif analysis of the CDPK protein sequence of *Saussurea involucrate.* The conserved motifs of SikCDPK proteins were discovered using the online searching tool MEME (http://memesuite.org/tools/meme). Different colors were used to indicate the conserved motifs.

The conserved motifs of snow lotus SikCDPK protein sequences were analyzed by MEME online software, and a total of 10 conserved motifs were found (**Figure 3**), of which motif 1, motif 2, motif 7, and motif 10 were present in all SikCDPK protein sequences. Except for c75948-g1-i1, which does not have motif 4 and motif 8, c86489-g1-i2 and c86622- g2-i1, which do not have motif 3, motif 5, motif 6, and motif 9, c88390-g1-i1, which does not have motif 6, and c86680-g3-i3, which does not have motif 6 and motif8, but has 2 motif7; c118412-g1-i1 does not have motif6, motif9, and motif3. Except for c118412-g1-i1, which does not have motif6, motif9, and motif3, the other 14 SikCDPK protein sequences have the same 10 conserved motifs, suggesting that the genes of the CDPK family of the snow lotus may be functionally similar.

### 3.4 Cloning and bioinformatics analysis of *SikCDPK1* gene

**Figure 4:**
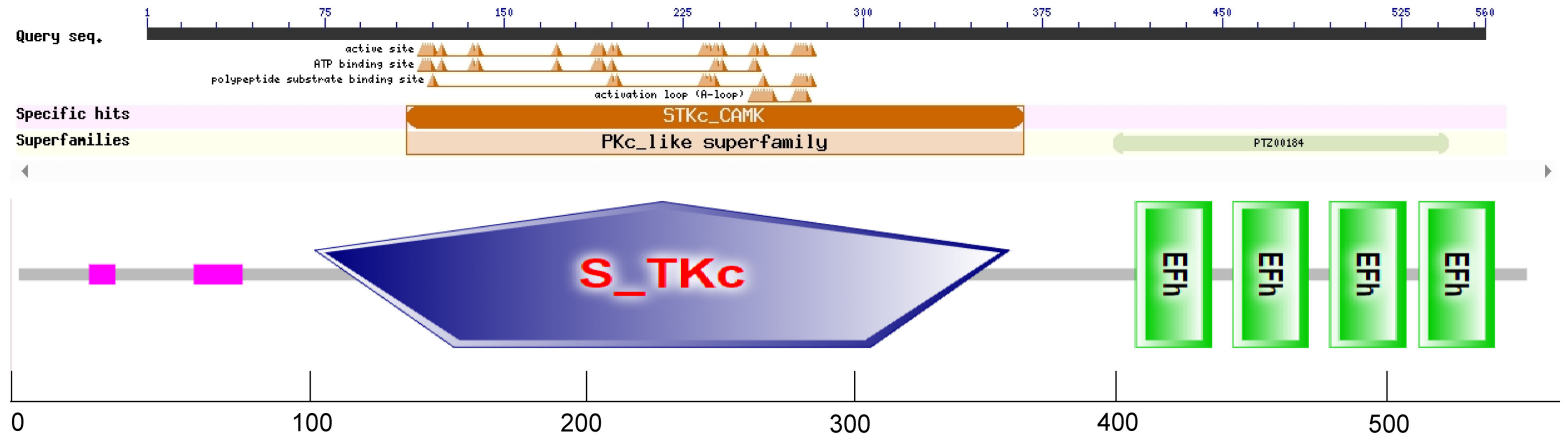
Domain organization of the *SikCDPK1*. The logos of domain organization were obtained from EMBL-EBI and SMART websites and were amended with Adobe Illustrator. The domains: V represents variable domain; K represents catalytic domain; I represents auto-inhibitory domain; C represents the region of calcium binding motifs: EF-hands.

After sequencing, we obtained the gene *SikCDPK1* with a coding region length of 1716 bp, and the GenBank accession number is KU133953. The conserved domains of *SikCDPK1* were analyzed by SMART online analysis tool, and the results showed that *SikCDPK1* has typical CDPK conserved domains, i.e., Ser/Thr protein kinase domains at the *N*-terminal end, a linker region in the middle, and a regulatory region at the *C*-terminal end (which is also a calcium-binding region), and there are four EF-hands structural domains, and it belongs to the *CDPK* gene family (**Figure 4**).

### 3.5 Tissue differential expression

**Figure 5:**
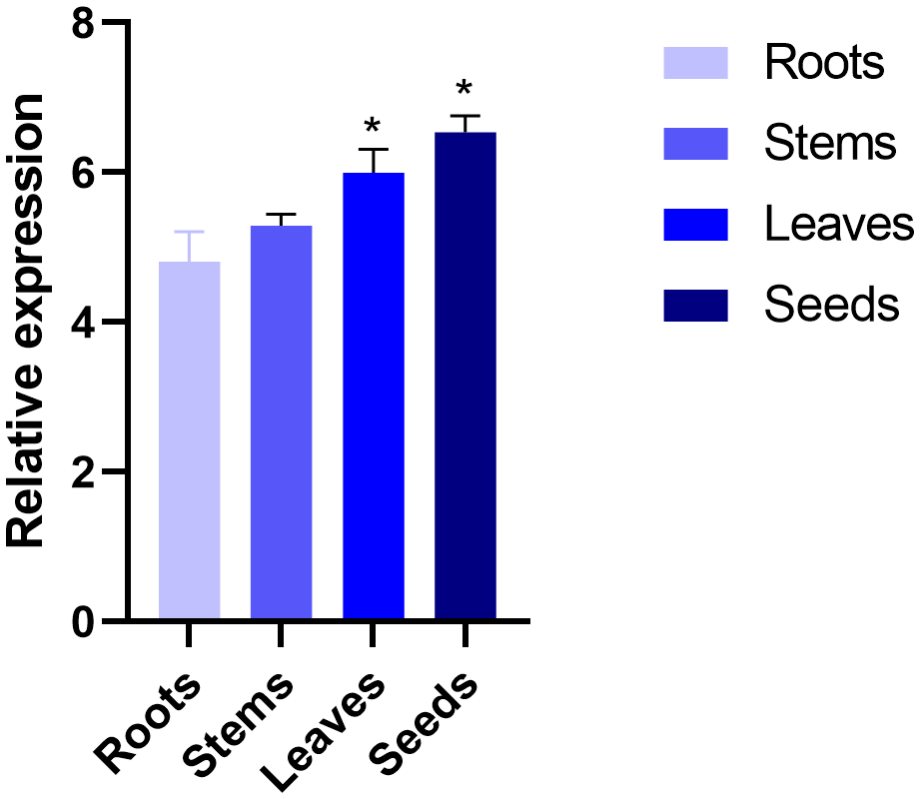
Tissue specific expression of *SikCDPK1* gene. Quantitative PCR data are represented as mean ± SEM. *p < 0.05. All date represent mean values for three independent biological replicates.

The expression of *SikCDPK1* gene in roots, stems, leaves and seeds of snow lotus was explored by qRT-PCR. As shown in **Figure 5**, *SikCDPK1* gene had the highest expression in seeds, others were leaves and stems in order, and the lowest expression was in roots. The gene expression in seeds, leaves compared with that in stems and roots reached significant differences. It indicates that *SikCDPK1* gene is present in snow lotus seeds, leaves, stems and roots, but there are differences in expression in different organs.

### 3.6 Analysis of gene expression induced by low temperature, ABA, GA_3_ and SA

**Figure 6:**
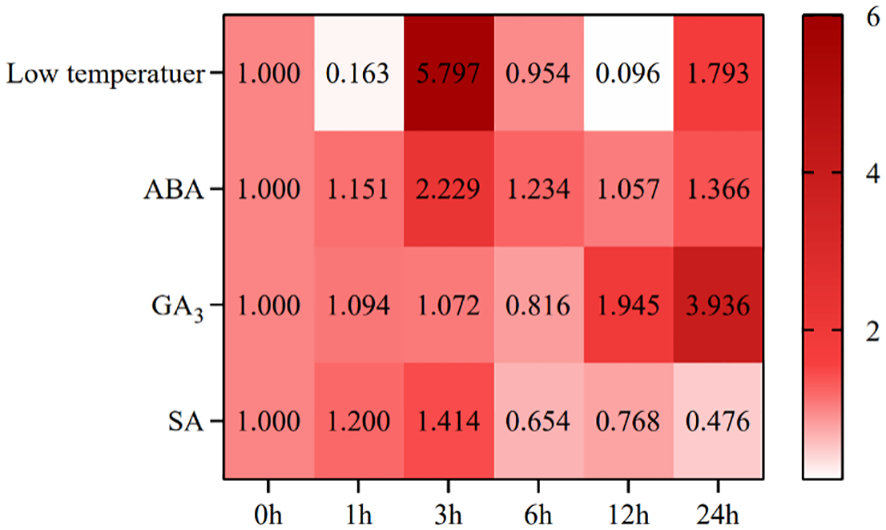
Expression profiles of *SikCDPK*1.

After low temperature, the expression of *SikCDPK1* showed the trend of “decline-rise-decline-rise” (**Figure 6**), which was 0.16 times of CK at 1h, and then reached the maximum at 3h, with the expression amount 5.8 times of CK, which reached the level of highly significant difference compared with CK. At 6h, it reached the initial level, and then dropped to the minimum expression amount at 12h. At 24h, the expression level started to rise again, which was 1.79 times of CK.

After ABA, the expression of *SikCDPK1* showed a “rising-declining-slowly rising” trend (**Figure 6**), reaching a peak at 3h, with an expression amount 2.23 times of CK, and reaching a highly significant difference compared with CK. At 12h, it reached the bottom, and the expression amount at the bottom was 1.057 times of CK, and then increased again at 24h, and the expression amount was 1.366 times of CK.

After GA_3_, the expression of *SikCDPK1* showed a trend of “slightly increasing-decreasing-increasing” (**Figure 6**), reaching the peak at 6 h, and the expression at the bottom of the peak was 0.816 times of CK, and then continued to increase. At 12h, the expression was 1.945 times of CK. It reached the peak at 24h, the expression amount was 3.9 times of CK, and reached a highly significant difference level compared with CK.

After SA, the expression pattern of *SikCDPK1* showed an overall “rising-declining” trend (**Figure 6**), reaching a peak at 3h, with an expression amount 1.4 times that of CK, which was significantly different from that of CK. After that, the expression continued to decline, and the lowest expression was at 24h, which was 0.476 times that of CK, which was significantly different from that of CK.

The above indicates that the expression of *SikCDPK1* gene was not only affected by low temperature, but also by ABA, GA_3_ and SA.

### 3.7 Promoter cloning and activity analysis of *SikCDPK1* gene

**Figure 7:**
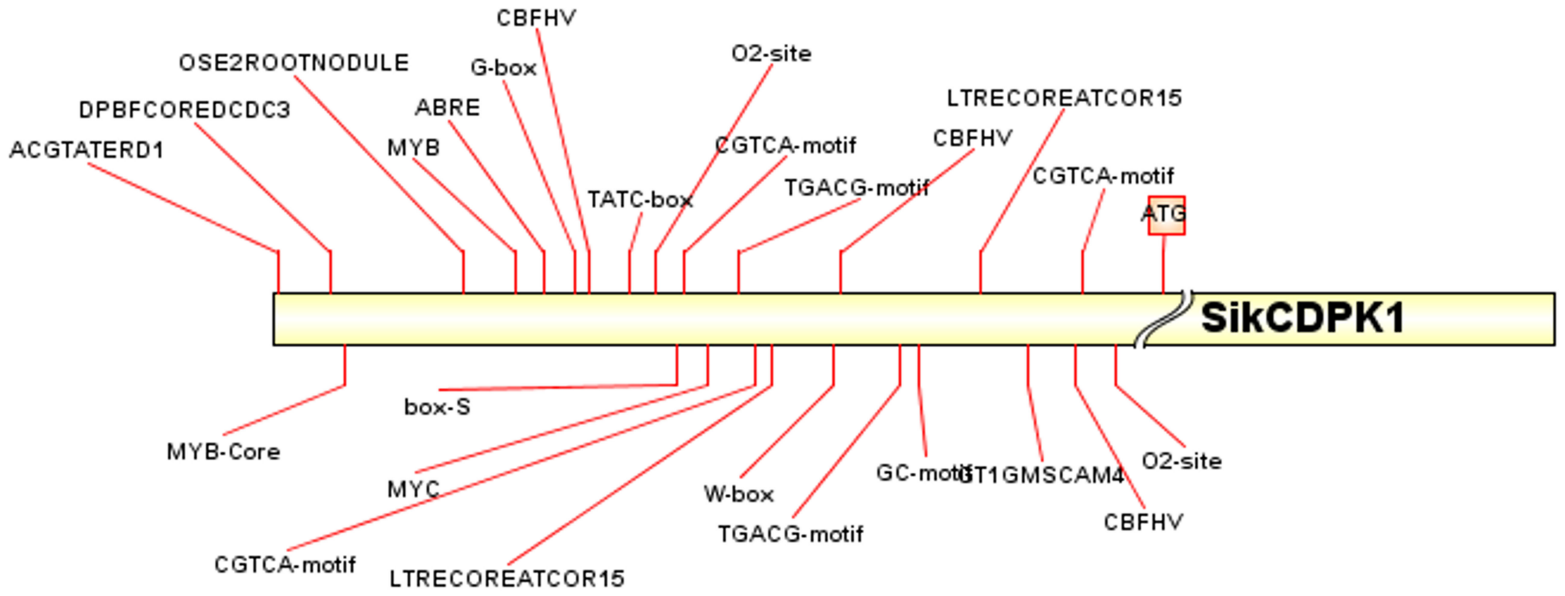
The main cis-regulatory element distribution of the promoter of *SiKCDPK1* gene. ATG is the start of the gene; Promoter elements to the left of the ATG are labeled with a red line.

**Figure 8:**
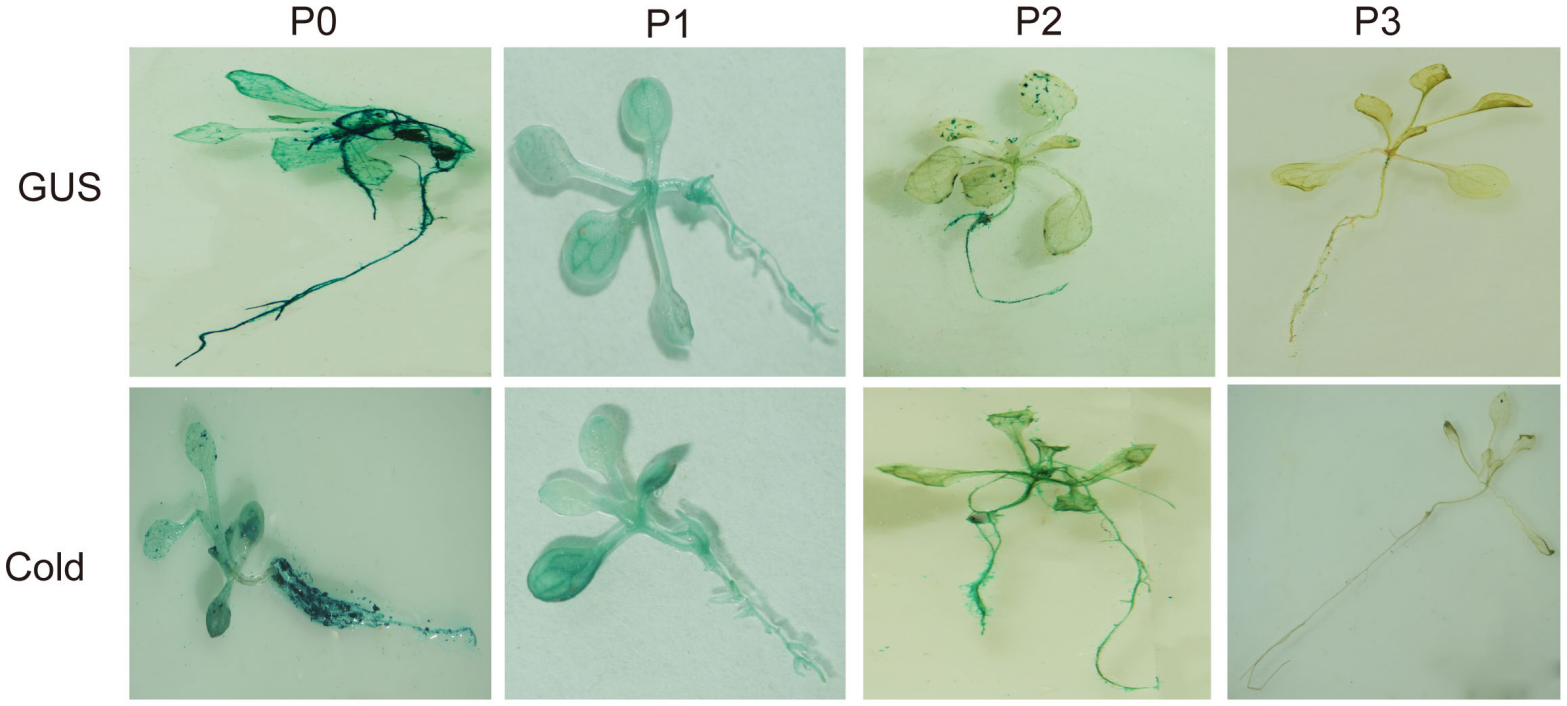
Histochemical assay for GUS expression in transgenic *A. thaliana*.

Analysis of the cloned *SikCDPK1* gene promoter obtained using PlantCARE revealed that the sequence contained several CAAT box and TATA box conserved elements, and also included abiotic stress-responsive homeopathic action elements: response to dehydration-responsive element MYB-Core, CBFHV with ACGTATERD1, salt-induced response element GT1GMSCAM4, cold-induced response element LTRECOREATCOR15, and light-responsive element G-box; cis-acting elements in response to hormone-induced stresses: methyl jasmonate-responsive elements CGTCA-motif and TGACG-motif, abscisic acid-responsive element DPBFCOREDCDC3, ABA-responsive element ABRE, gibberellin-responsive element TATC-box, etc. (**Figure 7**). In order to deeply investigate the function of *SikCDPK1* promoter, based on the full-length promoter P0, three deletion fragments responding to different stresses were designed according to the distribution of cis-regulatory elements, named P1, P2, and P3, respectively. Seedlings of GUS transgenic homozygous plants driven by the P0 promoter and its different 5’ end deletion fragments were stained with GUS histochemistry (**Figure 8**), and it was found that *Arabidopsis thaliana* with the transversion of the full-length sequence P0 was the most darkly colored, and the strongest coloring was found in the roots, stems, and leaves of the seedlings. The trans-promoter P1 fragment was relatively weakly colored, the trans-promoter P2 fragment was even more inferior, and the *Arabidopsis* seedlings with the trans-promoter P3 fragment did not show any coloration, indicating that the promoter activity of p*SikCDPK1* was from strong to weak as P0> P1> P2> P3. The color of promoter P2 was darkened and GUS activity was enhanced after low-temperature treatment. This is because the promoter sequence contains low-temperature inducible elements such as LTRECOREATCOR15 and CBFHV.

**Figure 9:**
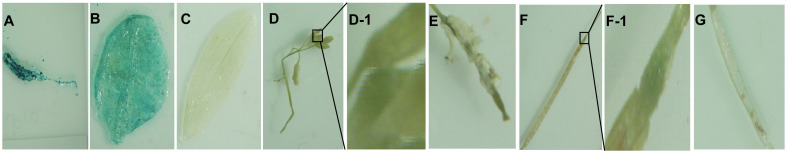
GUS assay results of different tissues of *A. thaliana* harboring *SikCDPK1* promoter. **(A)** Root, **(B)** Rosette leaf, **(C)** Stem leaf, **(D)** Flower and stem, (D-1). Magnification of **(D, E)** Petal, **(F)** Silique, (F-1). Magnification of **(F, G)** Seed.

GUS histochemical staining of GUS transgenic homozygous plants driven by the full-length fragment of the trans-P0 promoter revealed that GUS expression was detected mainly in roots (**Figure 9A**), rosette leaves (**Figure 9B**) floral pods (**Figure 9D**), and fruit stalks at the attachment of the angiosperm (**Figure 9F**), with higher expression in roots and rosette leaves, but not in suprachordate leaves (**Figure 9C**), petals (**Figure 9E**), young fruits, mature angiosperms, and seeds (**Figure 9G**), suggesting that the promoter of the *SikCDPK1* gene has a clear tissue expression specificity and exerts its expression-regulatory role mainly in roots and rosette leaves.

### 3.8 The overexpression of *SikCDPK1* improves low temperature tolerance of tobacco

**Figure 10:**
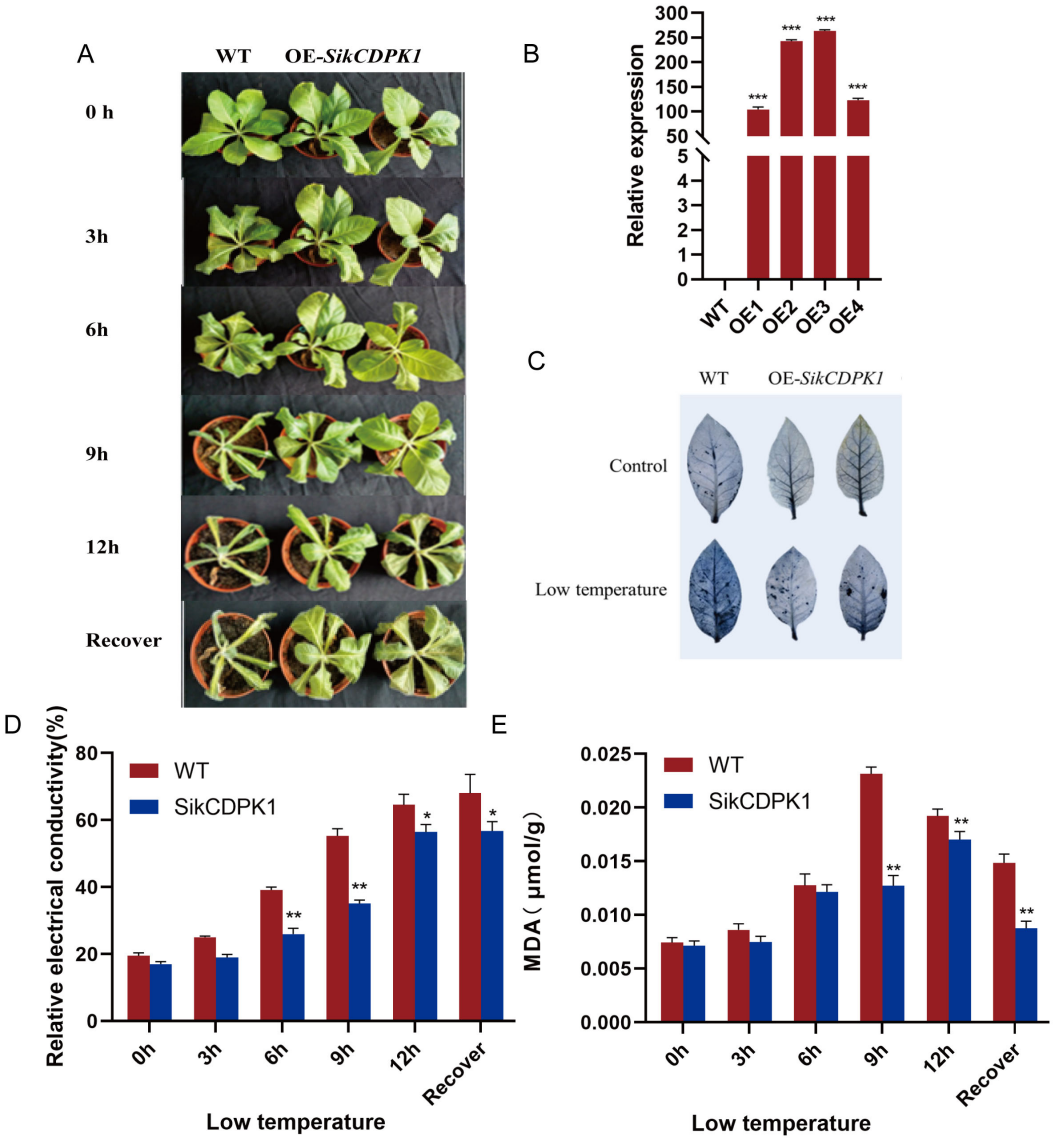
Overexpression of *SikCDPK1* improves cold tolerance of Tobacco. **(A)** The phenotypes of wild type tobacco and transgenic lines after low temperature for 0h、3h、6 h、9 h、12 h and recover. Bars =1 cm. **(B)** The expression level of overexpressed-*SikCDPK1* in Tobacco. wild type Tobacco; OE1, OE2, OE3 and OE4. **(C)** NBT staining of wild type tobacco and OE-*SikCDPK1* tobacco under low temperature. **(D)** Relative electricity conductivity. **(E)** MDA concentrations. Statistically significant differences compared to wild type. Values represent the mean ± SE from three biological replicates. *P< 0.05, **P< 0.01, ***p<0.001.

The wild type and transgenic tobacco lines were put into the -4°C artificial climate chamber for low temperature stress treatment. As shown in **Figure 10A**, there was no obvious phenotypic difference between the wild type and transgenic strains at room temperature; after 3 h in the incubator at -4°C, the leaves of wild type tobacco slightly drooped, which showed the characteristics of mild cold injury, and there was no obvious phenotypic change in the transgenic tobacco; after 6h of low temperature stress, the symptoms of cold injury of the wild type tobacco were more and more obvious, with the leaves moderately wilting and appearing as spots, while the transgenic tobacco did not have any obvious change. After 9h of low temperature stress, the wild type tobacco was basically completely wilted and waterlogged, with only the top leaflet buds showing signs of life, while the leaves of the transgenic tobacco were slightly weakened at this time; after 12h of low temperature stress, the wild type tobacco was completely wilted, and the leaves of the transgenic tobacco were curled up and drooping, which was a severe form of wilting. After 24h of room temperature recovery, there was no change in the wild-type tobacco, while the leaves of the transgenic tobacco began to stretch. High-level expression of SikCDPK1 gene in transgenic tobacco detected by real time fluorescence quantitative PCR (qRT-PCR) assay (**Figure 10B**).

Under normal growth conditions, there was no obvious difference between the leaves of wild type tobacco and transgenic tobacco, but the transgenic tobacco formed blue-black compound compared with the wild type tobacco plants after low temperature stress treatment, indicating that the transgenic tobacco lines accumulated relatively less ROS under low temperature stress conditions (**Figure 10C**).

The relative electricity conductivity and MDA activity of wild type and transgenic tobacco were measured at different treatment times under low temperature stress, and it was found that the relative electricity conductivity of both wild type and transgenic tobacco showed an obvious increasing trend with the extension of the stress time, and the relative conductivity of transgenic tobacco began to decrease after 1 day of recovery at room temperature, while the wild type still maintained an increasing trend; the relative electricity conductivity of transgenic plants was significantly lower than that of wild type during the whole process. The relative electricity conductivity of the transgenic plants was significantly lower than that of the wild type throughout the whole process, and reached a highly significant level at the 6h and 9h. This indicated that ion leakage caused by low temperature is related to injury of the cell membrane and its dysfunction. Thus lower ROS accumulation in transgenic tobacco causes lower accumulation of MDA, which suggests less damage to the cell membrane, so its integrity is better preserved. Hence the lower value of relative electrical conductivity (**Figure 10D**).

Before low temperature treatment, there was little difference between the MDA contents of wild type and transgenic tobacco, and with the extension of low temperature stress, the MDA contents of both wild type and transgenic tobacco showed an increasing trend, and the MDA contents of transgenic plants were lower than those of wild type, and reached a highly significant level at 9h. The MDA contents began to decrease at the recovery stage of room temperature. It indicated that the cell membrane of transgenic plants was less damaged under low temperature stress (**Figure 10E**).

## 4 Discussion

### 4.1 The identification and analysis of CDPKs in *Saussurea involucrata*

*CDPK* genes have been found in several plant genomes, such as *Arabidopsis* (Cheng et al., 2002), rice (Ray et al., 2007), and barley (Yang et al., 2017). In this study, we searched the transcriptome database of snow lotus based on conserved structural domains and identified 20 CDPKs in snow lotus. 34, 31, and 28 CDPKs were found in *Arabidopsis thaliana*, rice, and barley, respectively, which were much higher than those found in snow lotus (Cheng et al., 2002). A phylogenetic tree was constructed to categorize snow lotus CDPKs into four major subfamilies, which were consistent with *Arabidopsis* subfamilies. However, each subfamily in snow lotus has fewer members than in *Arabidopsis*.

The conduction of Ca^2+^ in *CDPK* genes is mainly through EF-hands, as well as the activation threshold of Ca^2+^ may be related to the differences in the number and location of EF-hands (Klimecka and Muszyńska, 2007). Generally, *CDPK* genes in plants have four EF-hands; however, there are exceptions for some *CDPK* genes in *Arabidopsis* and maize, which contain two or three EF-hands (Cheng et al., 2002; Kong et al., 2013). In our study, all SikCDPKs (except c75948_g1_i1) contained four EF-hands (**Table 2**). Therefore, the biological functional differences between c75948_g1_i1 (one EF-hand) and other SikCDPKs (four EF-hands) can be further explored.

CDPKs are widely distributed in plants (Simeunovic et al., 2016). Many members of the *CDPK* family play important roles in plant response to abiotic stress. *OsCDPK1* regulates rice salt and drought tolerance (Ho et al., 2013). The *CDPK* family is involved in drought or salt stress adaptation by inducing ABA responsive genes and adjusting the ABA-induced anionic channel (SLAC1, SLAH3), leading to stomata adjustment in *Arabidopsis* (Edel and Kudla, 2016; Zhang et al., 2020). Overexpression of *MDCPK1A* in tobacco removes ROS accumulation and regulates the expression of stress-related genes, thereby significantly increasing cold and salt resistance (Dong et al., 2020). Excessive expression of *ZMCPK1* in corn leaf inhibits the expression of cold inducing marker gene *ZMERF3*. The ectopic expression of *ZMCPK1* in *Arabidopsis* reduced the adaptation of the plant in terms of cold resistance, indicating that *ZMCPK1* acts as a negative regulatory factor under cold stress (Weckwerth et al., 2015). *Arabidopsis CPK28* functions as a negative regulator of an immune signal which responds to immune responses by regulating *BIK1* (multi-mode identification receptor (PRR)) (Simeunovic et al., 2016). *CDPK* can also be used as a negative regulator of the stress response. Transgenic plants overexpressing *CDPK* are more sensitive to abiotic stress and biotic stress. *Arabidopsis cpk23* mutant increases tolerance to drought and salt stress, but resistance to drought and salt stress is reduced in *AtCPK23* overexpressing plants (Ma and Wu, 2007). Taken together, it is apparent that CDPKs participate in plant abiotic and biotic stress in the form of positive and negative regulation. Virus-induced silencing of *CDPK2* and *CDPK3* showed that *CDPK1* and *CDPK2* are involved in the regulation of *AVR9/CF-9* genes, and *CDPK* is a necessary condition for plant *AVR9/CF-9* induced hypersensitivity during pathogen infection (Romeis et al., 2001). *CDPK10* in maize is also involved in defense signaling pathways (Murillo et al., 2001).

The specificity of signaling can be determined by the subcellular location of CDPK proteins, which can be regulated at the translational and post-translational levels. Predictions indicate that SikCDPKs are mainly located in several cellular compartments, namely chloroplasts, nucleus, and cytoplasm. The distribution of SikCDPKs in different cellular compartments suggests that there are also differences in signaling specificity. It was also predicted that most SikCDPKs contain *N*-myristoylation sites, and *CDPK* genes with *N*-myristoylation motifs can promote protein-membrane and protein-protein interactions (Lu and Hrabak, 2013; Martín and Busconi, 2000). Myristoylation may be part of a major signaling process that directs these proteins to cell membrane binding sites of the 19 SikCDPKs identified in snow lotus, all contain palmitoylation sites. Palmitoylation sites may provide important clues for subcellular localization (Martín and Busconi, 2000).

Sequence motifs are becoming increasingly important in gene regulation analysis. In this study, we found the similarity in the distribution of motifs in the SikCDPKs family by analyzing motifs. By resolving sequence motifs, functions in biological processes can be resolved. When a large number of repetitive sequence structures appear in a certain large class of sequences, it is valuable to explore its significance (Bailey et al., 2015). Therefore, in the subsequent process of exploring the functions of SikCDPKs, the biological significance involved in each biological process can be further understood based on motifs.

### 4.2 *SikCDPK1* plays a positive role in conferring low temperature tolerance

A new calcium-dependent protein kinase gene, *SikCDPK1*, was cloned from snow lotus, and the real-time quantitative PCR results showed that the expression of the *SikCDPK1* gene was induced by low temperature stress, which was similar to the results of the previous studies on *PeCPK10* and *OsCPK13* (Chen et al., 2013; Wan et al., 2007).

To further investigate the function of *SikCDPK1*, overexpressing *SikCDPK1* was found to have no significant difference between the transgenic plants and the wild-type phenotype. This is similar to the findings of *OsCPK13*, suggesting that the *SikCDPK1* gene has little effect on the growth status of tobacco. Low-temperature stress was applied to tobacco trans-*SikCDPK1* gene, and the transgenic plants were significantly better than the wild type in terms of low temperature tolerance. This was similar to the results of overexpression of *AtCDPK1*, *PeCPK10*, *OsCPK13* and *VaCPK20* genes (Atif et al., 2019; Chen et al., 2013; Wan et al., 2007; Dubrovina et al., 2015), and the transgenic plants significantly improved the plant resistance to low temperature. Measurement of physiological indexes in *SikCDPK1*-transgenic tobacco under low temperature stress revealed that the MDA content and conductivity were lower than those of the wild type plants. Plant cells produce excess of reactive oxygen species (ROS), such as H_2_O_2_, O^2-^ and -OH, and the accumulation of MDA were subsequently found (Upadhyaya and Khan, 2007). The high expression of ROS could result in the deep damage of plants, such as lipid peroxidation and electrolyte leakage (Mishra et al., 2009). Notably, MDA produced in membranes could destroy membrane structure and cause protein degradation, and then it aggravated by the damage of low temperature (Martinez et al., 2018; Petrov et al., 2015). For keeping the balance of ROS numbers, plants had evolved some enzymatic and non-enzymatic antioxidant systems to eliminate or reduce the ROS damage induced by low temperature stress (Wani et al., 2018; Wang et al., 2016). Thus it can reduce the damage of the plasma membrane caused by low temperature, and make the transgenic plants significantly increase the ability to tolerate low temperature.

Ca^2+^ is an important second messenger in the low-temperature signaling pathway (Monroy and Dhindsa, 1995). When *Arabidopsis thaliana* or *Medicago sativa* (alfalfa) face low temperature stress, Ca^2+^ from extracellular stores flows into the cells and causes a rapid increase in the cytoplasmic Ca^2+^ content. This transient increase in Ca^2+^ concentration is necessary to induce the expression of cold-adapted genes and cold tolerance in cold-resistant plants (Cao et al., 2023). In *Arabidopsis*, the full expression of some cold-regulated genes, such as the CRT/DRE-controlled cor6.6, is dependent on an increase in Ca^2+^ levels (Thomashow, 1999). When Ca^2+^ chelating agents - BAPTA, or Ca^2+^ channel blockers -La^3+^, inhibited the low-temperature-induced Ca^2+^ input, the expression of low-temperature induced *cas15* gene was attenuated, and the cold-adapted genes were not expressed at the same time (Cao et al., 2023). Gene expression was reduced, and the cold acclimatization ability of alfalfa was reduced. Therefore, it is speculated that it may be that the inhibition of Ca^2+^ input with the prolongation of low-temperature treatment affected the expression of the *SikCDPK1* gene, resulting in the incomplete up-regulation of *SikCDPK1*.

### 4.3 Demonstration of *SikCDPK1* promoter activity in transgenic plants

Promoters are important regulatory elements that determine the temporal and spatial order of gene expression to a certain extent, and the study of promoter activity and function can help to investigate the function and expression regulation mechanism of genes in depth. In order to understand the expression regulation of snow lotus *SikCDPK1* gene in response to adversity, our group cloned the promoter sequence of the *SikCDPK1* gene by TAIL-PCR (Shi et al., 2022). The promoter sequence prediction revealed that the promoter region contains not only the TATA-box and CAAT-box (Alok et al., 2020), but also MYB-core、ACGTATERD1、LTRECOREATCOR15、CBFHV and other cis-acting elements related to low temperature. Transient expression of GUS or other marker genes has been used in assessing activity and tissue specificity of plant promoters (Basu et al., 2003). *SikCDPK1* promoter P0 can promote the expression of GUS gene under low-temperature stress, and it has strong expression specificity in different tissues. The *SikCDPK1* promoter has a promoter activity center more than 480 bp upstream of the ATG start codon, and the promoter P0 and P2 is able to be induced by low temperature. Interestingly, the *SikCDPK1* gene had the lowest expression in roots, while the *SikCDPK1* promoter had the highest activity in roots. This may be the receptors are different and the gene regulatory mechanisms have changed, resulting in inconsistent expression (Hernando et al., 2017).

Although the results of this experiment showed that the *SikCDPK1* gene significantly improved the low temperature tolerance of transgenic tobacco, the transcriptome database of snow lotus was still incomplete, and the chromosomal mapping results were not clear, so it was difficult to find and name more SikCDPKs gene family members. In the future, the SikCDPKs family genes related to stress resistance can be mined from the complete snow lotus transcriptome database, and the function, gene regulation of the genes can be studied in depth. These studies will provide a theoretical basis for exploring the upstream regulatory sequences of the *SikCDPK1* gene and analyzing the regulatory mechanism of the *SikCDPK1* gene in response to adversity.

## 5 Conclusions

In this study, 20 *CDPK* genes with conserved motifs that were significantly similar to those of *Arabidopsis thaliana* and rice were identified from snow lotus, and these SikCDPKs could be divided into four subgroups. Among them, *SikCDPK1* responds to low temperature and hormonal treatment, and is expressed differently in different organs of snow lotus. The promoter of *SikCDPK1* gene contains a cold induction element, and the promoter activity is increased after low temperature. The degree of damage in overexpressing *SikCDPK1* plants was lower after low temperature treatment, the ROS accumulation was less, and the relative conductivity and MDA content changed slightly. The results indicated that *SikCDPK1* could positively regulate the low temperature tolerance of plants by activating the cold-inducible element in the promoter, improving gene expression, maintaining cell membrane stability, and reducing the accumulation of reactive oxygen species. Our study demonstrated that *SikCDPK1* positively regulates cold tolerance, providing a new genetic resource for genetic improvement of low temperature tolerance in snow lotus. Future studies should continue to improve the construction of a transcriptome database for the *CDPK* family in snow lotus. Investigating the signaling network and biochemical functions of *SikCDPK1* to gain a deeper understanding of its molecular mechanism in regulating low temperature stress tolerance.

## Data Availability

The original contributions presented in the study are included in the article/**Supplementary Material**. Further inquiries can be directed to the corresponding author.
