# Supplementary figures and images for: Identification of SikCDPK family genes to low-temperature by RNA-seq approaches and functional analysis of SikCDPK1 in Saussurea involucrata (Kar. & Kir.)
Source: Front Plant Sci. 2024 Sep 30;15:1436651. doi: 10.3389/fpls.2024.1436651 (PMC11471645; doi:10.3389/fpls.2024.1436651)

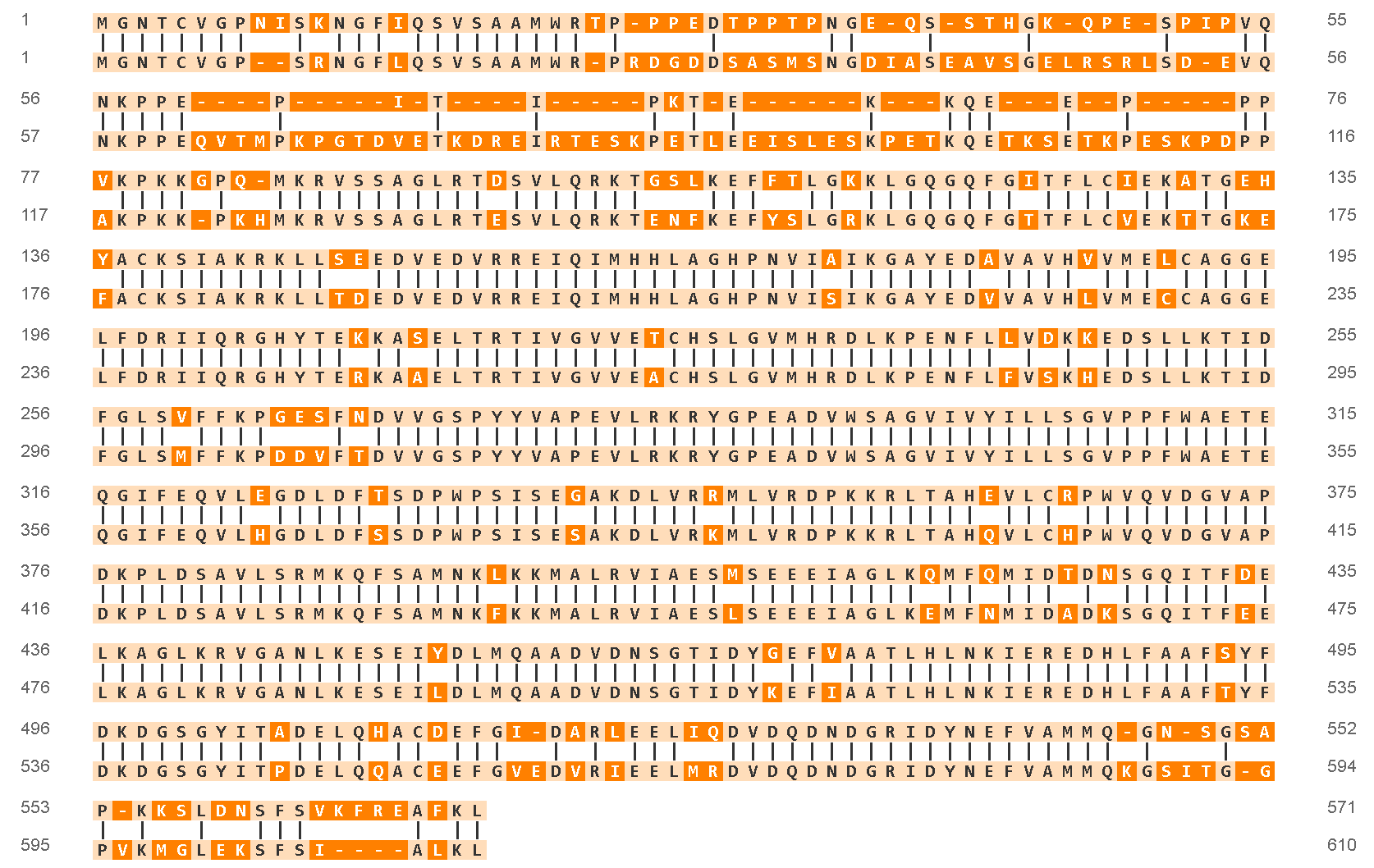

Supplement: Supplementary file 2 [file Image1.png]

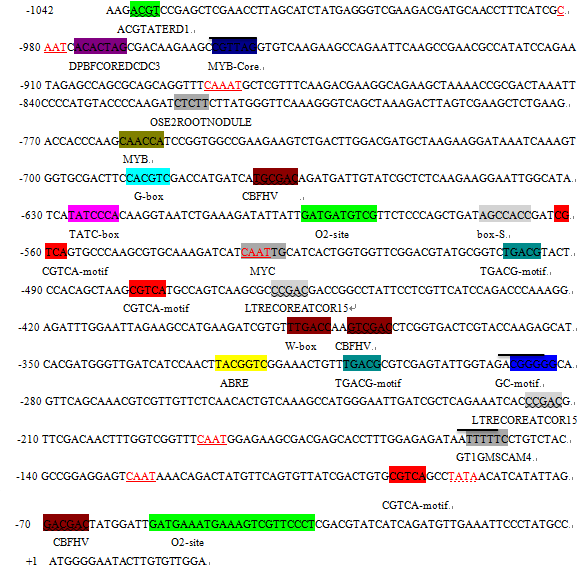

Supplement: Supplementary file 3 [file Image2.png]

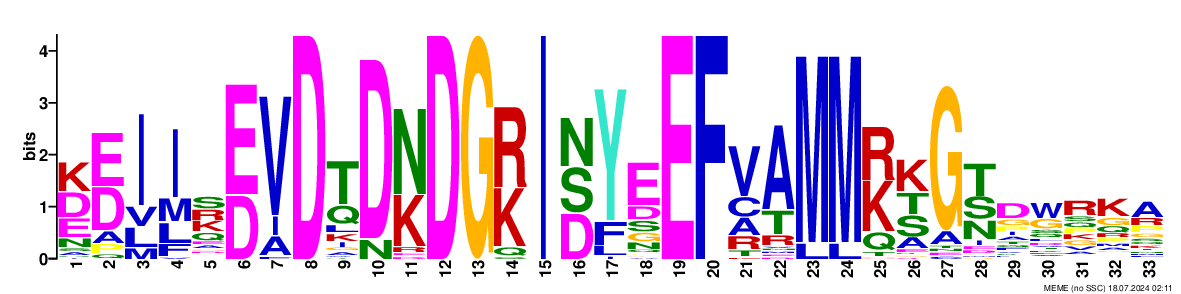

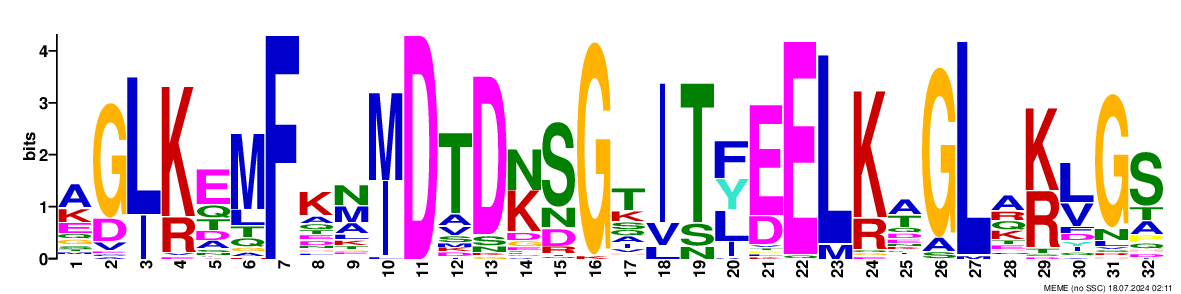

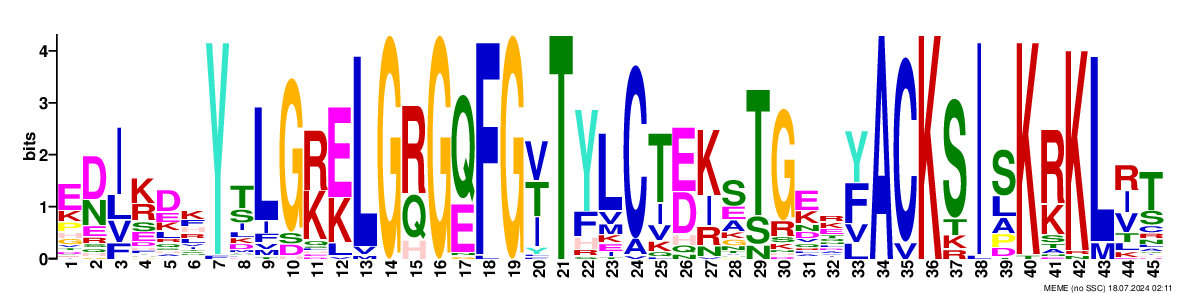

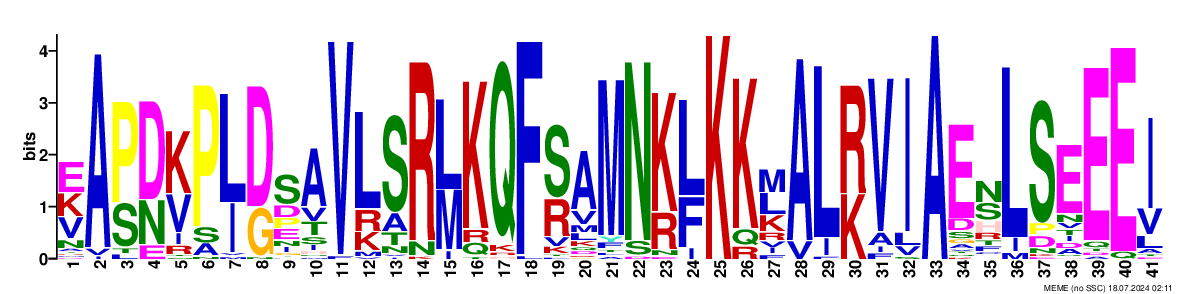

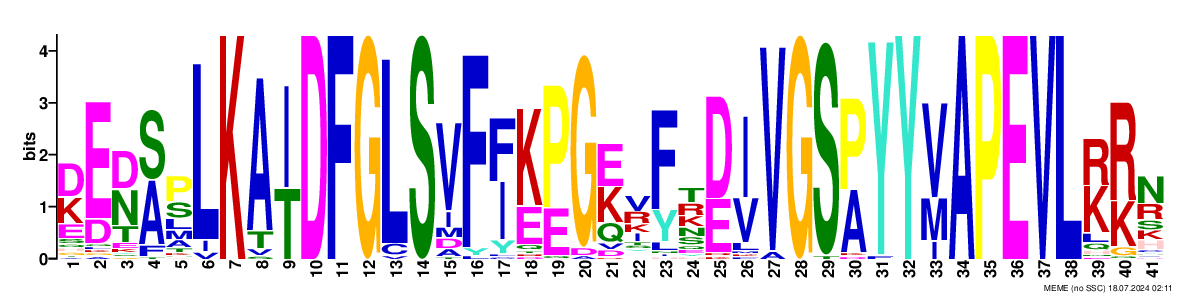

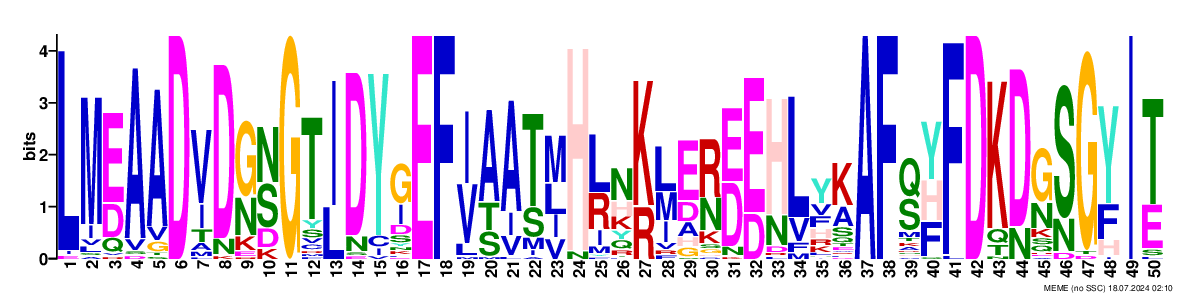

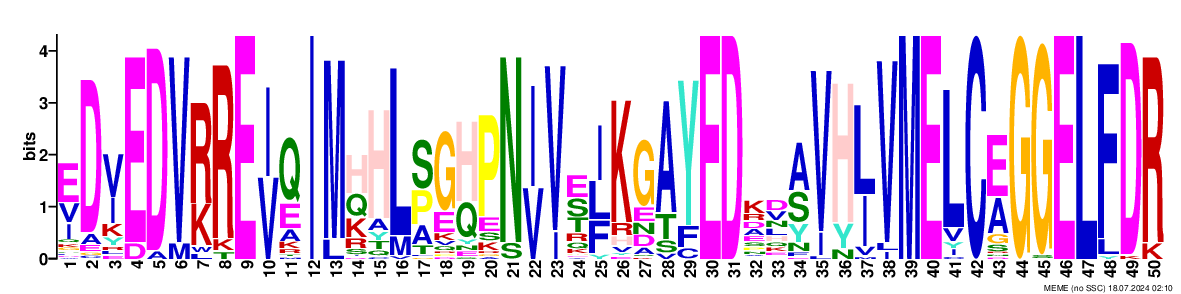

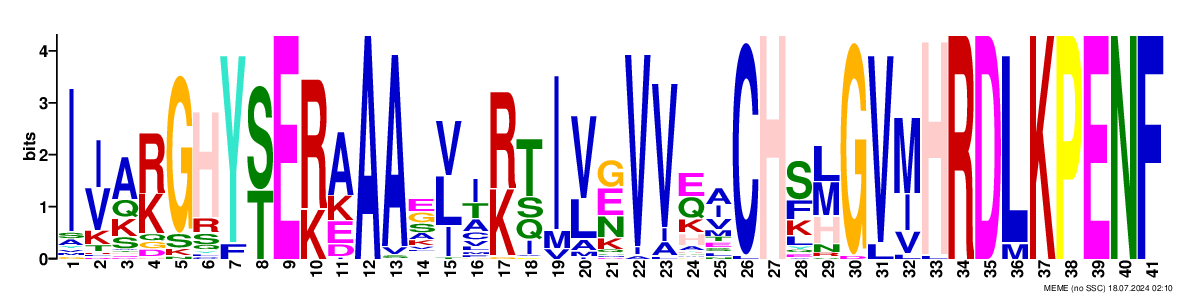

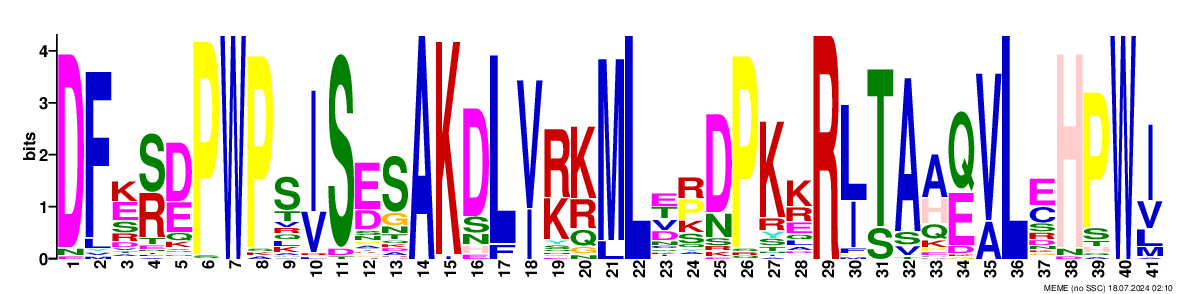

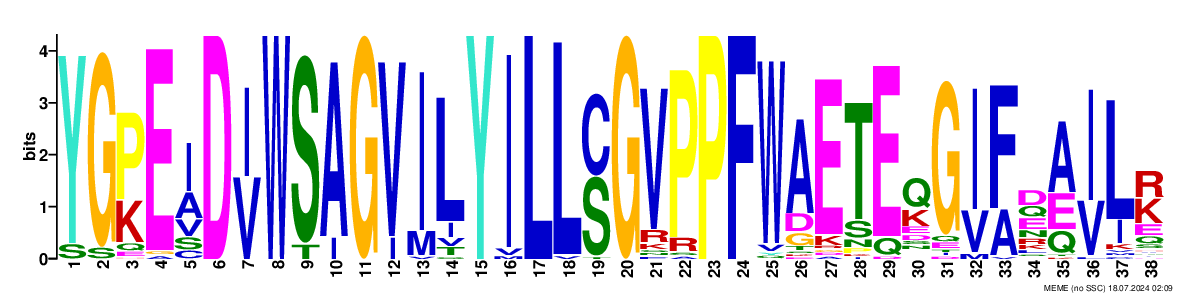

Supplement: Supplementary file 4 [file Table1.docx]
